# Enhanced virulence of *Fusarium* species associated with spear rot of oil palm following recovery from osmotic stress

**DOI:** 10.1080/21501203.2017.1336497

**Published:** 2017-06-09

**Authors:** Suwandi Suwandi, Seishi Akino, Norio Kondo

**Affiliations:** a Laboratory of Plant Pathology, Faculty of Agriculture, Sriwijaya University, Palembang, Indonesia; b Laboratory of Plant Pathology, Division of Bioresources and Product Science, Research Faculty of Agriculture, Hokkaido University, Sapporo, Japan

**Keywords:** *Fusarium*, enhanced virulence, weak pathogen, *Elaeis guineensis*, crown disease, osmoadaptation

## Abstract

*Fusarium* spp., which are common inhabitants of oil palm leaves, are weak pathogens of common spear rot (CSR). We investigated the influence of osmotic stress on the growth, virulence, and activity of cell wall-degrading enzymes of CSR fungi, using potato dextrose agar (PDA) supplemented with KCl or sucrose (hyperosmotic medium). Hyperosmotic stress significantly inhibited mycelial growth, but growth rapidly recovered when mycelia were transferred to control medium. When inoculated into oil palm spear leaflets, *Fusarium* sp., and *F. incarnatum* precultured on 1.0 and 1.5 M KCl-hyperosmotic medium induced lesions that were two to four times larger than those in non-stressed cultures, suggesting enhanced virulence of the weak pathogens. Lesion size was not greatly affected in hyperosmotic cultures of moderately virulent *F. sacchari*. No activity of pectin lyase was detected in liquid cultures of the *Fusarium* isolates. All isolates except *F. incarnatum* BT48 secreted polygalacturonase (PG), which was active in both liquid cultures and inoculated leaves. Significantly increased PG activity (5–32-fold) was observed on leaves inoculated with hyperosmotic cultures of *Fusarium* sp. and *F. sacchari*. These findings suggest that *Fusarium* sp., *F. incarnatum,* and *F. sacchari* exhibit an adaptive physiological plasticity to hyperosmotic stress that results in enhanced virulence.

## Introduction

Common spear rot (CSR), also known as crown disease, is the most common disease in young oil palm (*Elaeis guineensis* Jacq.) worldwide (Breure & Soebagjo 1991; Chinchilla et al. 1997; Corley & Tinker 2003). The disease has caused considerable losses of young oil palms, but few detailed studies of the pathogenic agent of the disease have been conducted. Our study (Suwandi et al., 2012) suggested that *Ceratocystis paradoxa, Fusarium sacchari*, and weakly virulent fungi dominated by *Fusarium* spp. were associated with oil palm CSR in Indonesia. *C. paradoxa* and *F. sacchari* are strongly and moderately virulent, respectively, but their prevalence was low. *Fusarium* sp. (an undescribed species belonging to the *Gibberella fujikuroi* species complex) and *F. incarnatum* were more abundant on both rotted and healthy spear leaflet tissues. *Fusarium* species are considered weakly virulent as they induce only a very small lesion under 5 mm in length on the normal oil palm leaves.

The genetic and environmental factors have been known to predispose oil palm leaves to the spear rot. Deli *dura*, a fast growing and widely used progeny is highly susceptible to the disease (Breure & Soebagjo 1991). Certain environmental stresses such as drought, poor drainage, root wounding, and high doses of fertilisers have been suggested as predisposing factors for susceptibility to CSR (Alvarado et al. 1996). Sterling and Alvarado (1996) reported that peaks of CSR incidence in the field were associated with the beginning of the rainy season. The onset of the rainy season and fertiliser application promote the rapid and succulent growth of oil palm leaves that are susceptible to fungal rotting (Alvarado et al. 1996; Suwandi & Kondo 2012). Periodic drought stress was necessary to promote the rapid and succulent growth of oil palm leaves (Suwandi & Kondo 2012).

Drought during the dry season and fertiliser application at the beginning of the rainy season might expose oil palms to salt and osmotic stress (Mutert et al. 1999). Drought reduces the leaf osmotic potential of oil palm seedlings (Suresh et al. 2010) and may also impose either a hyperosmotic shock (or osmotic upshift) or hyperosmotic stress on leaf-inhabiting fungi. The influence of drought stress on *Fusarium* spp. commonly found on spear leaf tissue is not known. Although growth *Fusarium* spp. is suppressed under osmotic stress (Ramirez et al. 2004; Marín et al. 2010; Palacios et al. 2014), little information is available on the responses of *Fusarium* spp. after recovery from stress.

Plant-pathogenic *Fusarium* species produce an array of hydrolytic enzymes that enable them to penetrate and infect host tissue (Kikot et al. 2009). Polygalacturonases (PGs) and pectin lyases (PNLs) are among the first enzymes produced during infection by *Fusarium* spp., and they play a crucial role in plant tissue penetration and colonisation (Aleandri et al. 2007; Mariotti et al. 2009).

In this study, we investigated the effects of drought and salt stress on the growth, virulence, and activity of pectinase enzymes of *Fusarium* sp., *F. incarnatum*, and *F. sacchari* from spear rot of oil palm.

## Materials and methods

### Isolates

Four isolates of *Fusarium* species associated with CSR of oil palm in Indonesia were used in this study: two of species from the *Gibberella fujikuroi* species complex (*Fusarium* sp. isolate BT41 and FP41), one of *F. incarnatum* (BT48), and one of *F. sacchari* (SJW1). The GenBank accession numbers of the partial translation elongation factor 1-alpha (EF-1α) of the isolates are HM770731, HM770732, HM770722, and HM770734 for isolates BT41, FP41, BT48, and SJW1, respectively. Three of the isolates were weakly virulent to susceptible spear leaf leaflets, whereas SJW1 was moderately virulent (Suwandi & Kondo 2012). Single-spore subcultures were maintained for routine use on synthetic nutrient agar (Nelson et al. 1983) slants at 25°C.

### Osmotic stress treatment, growth under stress, and recovery

Isolates were grown on a cellophane membrane (Toyo Roshi Co., Tokyo, Japan) over potato dextrose agar (PDA; Difco) supplemented with 0.6, 1.0, or 1.5 M KCl (KCl hyperosmotic medium) or 1.2 M sucrose (sucrose hyperosmotic medium) in 85-mm plastic Petri plates. Non-stressed cultures were grown on a cellophane membrane over PDA. The water potential of the medium was about – 0.35, – 3.35, – 4.85, – 7.04, and – 4.85 MPa for PDA and PDA supplemented with 0.6 M KCl, 1.0 M KCl, 1.5 M KCl, and 1.2 M sucrose, respectively, as predicted at 25°C following Bruehl and Kaiser (1996) and Cochrane and Cochrane (2009). Petri plates of the same isolate and osmotic potential were sealed in polyethylene bags. Triplicate sets of each treatment were incubated at 28°C for 8 days in the dark, and all experiments were repeated twice. The colony diameter of cultures was measured daily until the colony reached the edge of the plate. The radii of the colonies were plotted against time for each replicate, and linear regression was applied to obtain the mycelial growth rate (mm/day) as the slope of the line. To determine the mycelial growth rate during recovery, a mycelial strip (3 × 1 cm) was peeled off of the cellophane of hyperosmotic or PDA cultures and subsequently transferred to a pectin-amended malt extract agar (PMEA; 10 g L^–1^ malt extract, 10 g L^–1^ citrus pectin, 1 g L^–1^ peptone, 10 g L^–1^ gellan gum, 10 g L^–1^ agar) or minimal medium (MM; Correll et al. 1987).

### Pathogenicity test

Virulence was determined based on the length of the lesion produced on inoculated cut spear leaves of oil palm. Spear leaves with 30 leaflets (leaflets were not detached) were cut from 3-year-old oil palm cultivar Dumpy (Dy × P) growing in an experimental field of the Faculty of Agriculture, Sriwijaya University, Palembang, Indonesia. The leaves were collected during the normal growth period after 3 months of fertiliser application in the rainy season. A piece (3 × 3 mm) of mycelial strip was peeled from the cellophane after 8 days of growth on hyperosmotic media, washed with sterile water, and inoculated on the basal part of unopened leaflets. Prior to inoculation, a single puncture wound was made using a sterile syringe needle. Inoculated spear leaves were wrapped in a wet paper towel and clear polyethylene sheet to maintain humidity. The cut rachis of the inoculated spear leaf was inserted into a test tube (123 × 16 mm) filled with 3 mL sterile water. The test tubes with spear leaves were placed on a tube stand and incubated at room temperature (26–29°C) with diffuse light. The lesion length was measured 4 days after inoculation. Each mycelial strip was inoculated into 24–36 leaflets, and the experiment was repeated thrice. *Ceratocystis paradoxa* B116, a highly virulent CSR isolate, was the positive control in virulence assays.

## Enzyme extraction from *fusarium* isolate cultures

A mycelial strip (3 × 1 cm) was peeled from hyperosmotic or PDA cultures and subsequently grown in 8 mL liquid medium (10 g L^–1^ malt extract, 10 g L^–1^ citrus pectin, and 1 g L^–1^ peptone) in 85-mm Petri plates at 28°C in the dark. The culture medium was collected 1, 2, and 3 days later, centrifuged at 15,000 *g* for 10 min at 4°C, and dialysed against distilled water at 4°C. The resultant supernatants were used immediately or stored at 4°C for less than 3 weeks. Three replicate plates were used for each treatment, and the experiment was repeated twice.

### Extraction of pectolytic enzymes from inoculated spear leaf leaflets

PG and PNL activity in lesions of spear leaf leaflets inoculated with the 1.5 M KCl-hyperosmotic or PDA culture was determined 4 days post-inoculation. Freshly collected diseased tissues (0.2 g) were cut in small pieces and macerated in a pre-cooled mortar with quartz sand and 1 mL extraction buffer (50 mM Tris-HCl, pH 7.8, 1 mM PEG 6000, 1 mM PMPF, 8% polyvinylpyrolydone, 0.01% Triton X-100; Venisse et al. 2001). The homogenate was centrifuged at 5000 *g* for 20 min in a pre-cooled centrifuge. Debris-free supernatants were used immediately for plate assays, which were performed on each of six representative lesions. The experiment was repeated twice.

### Assay of pectinase activity

PG activity of culture supernatants and leaf homogenates was assayed using the agar diffusion procedure (Taylor & Secor 1988). The assay was run in 85-mm plastic Petri plates containing 15 mL 1% agarose, 0.1% polygalacturonic acid, 50 mM sodium acetate (pH 5.0), and 10 mM ethylenediaminetetraacetic acid (EDTA). The supernatants and homogenates (30 μL) were pipetted into 6-mm diameter wells punched in the agarose with a cork borer. After incubation at 28°C for 14 h, the gel was stained with 0.05% ruthenium red for 1 h, and the area of the clear zone of activity was calculated using ImageJ ver. 1.49c (NIH, Bethesda, MD, USA). PG activity was estimated based on a logarithmic regression curved (*R*^2^ = 0.996) obtained using standard PG (EC 3.2.1.15; Sigma P-9179).

PNL activity was determined using agar plate diffusion and spectrophotometric assay. The agar plate diffusion assay was performed as described earlier in the PG assay, except agarose medium containing 0.1% pectin (≥85 esterified; Sigma P9561), 100 mM pH 6.0 citrate-phosphate buffer, 1 mM EDTA, and 10 mM CaCl_2_ was used. The spectrophotometric assay was performed by monitoring the increase in absorbance at 235 nm due to the formation of unsaturated uronide product at 40°C. The assay mixture consisted of 200 μL filter-sterilised 0.4% citrus pectin solution in 100 mM citrate-phosphate buffer (pH 6.0) with or without the addition of 1 mM EDTA and 10 mM CaCl_2_ (Aleandri et al. 2007).

### Statistical analyses

One-way analyses of variance were performed to assess differences in mycelial growth rate and lesion length in each experiment. A Waller–Duncan *K*-ratio *t*-test was used to compare mycelial growth rate, lesion length, and PG activity of culture homogenate. Statistically significant differences between the PG activity of inoculated leaves were determined using paired *t*-tests. Analyses were implemented using the packages Rcmdr and Agricolae for R ver. 3.3.1 (R Foundation for Statistical Computing).

## Results

### Growth under osmotic stress and recovery

**Figure 1.**
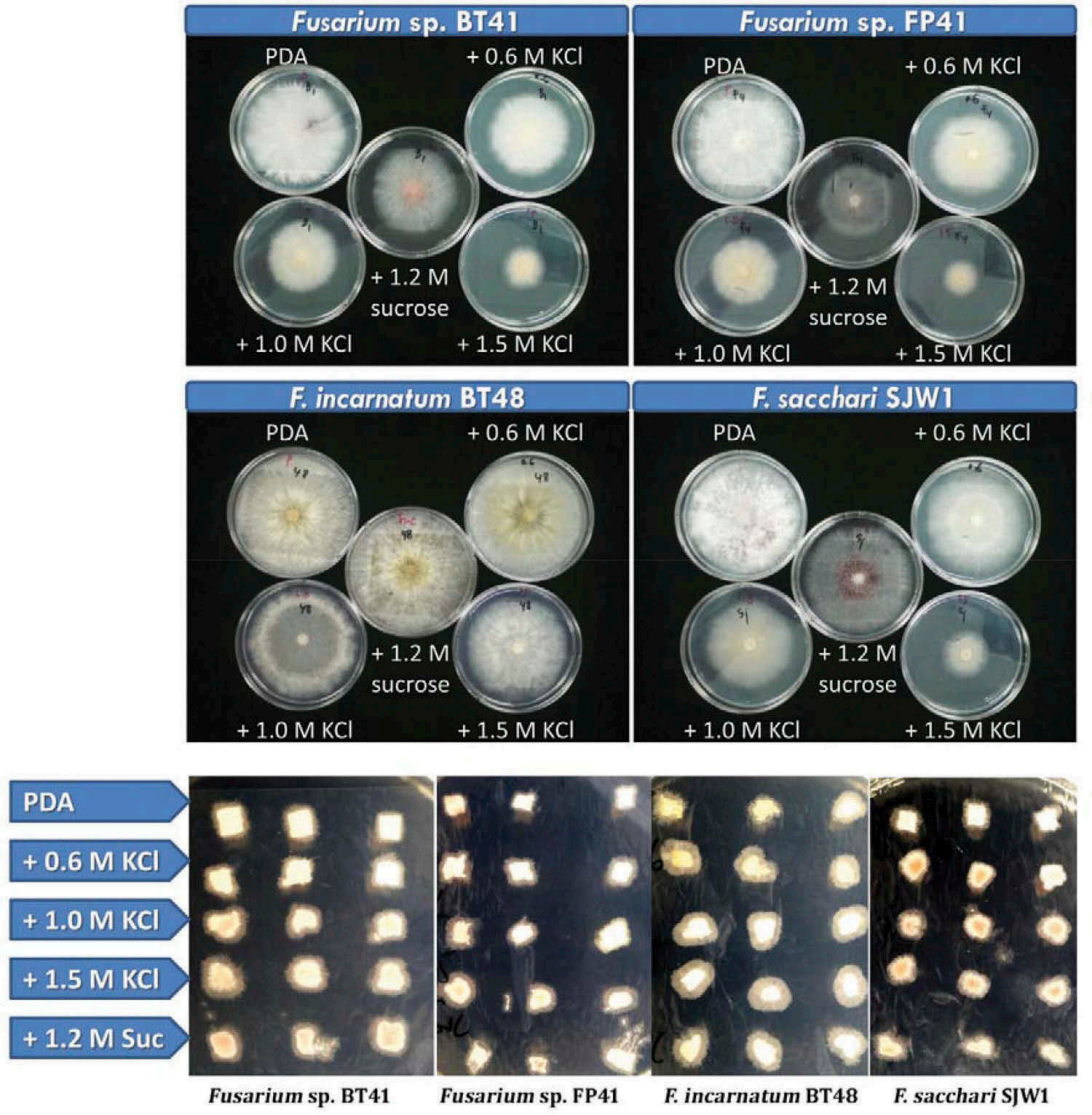
Growth of *Fusarium* isolates from spear rot of oil palm under hyperosmotic stress and recovery. The first and second rows show 8-day-old cultures under stress, and the bottom row shows the growth of mycelial disks after 12 h transferred onto cellophane-overlaid minimal medium (MM). PDA supplemented with 0.6 M KCl (–3.35 MPa), 1.0 M KCl (–4.85 MPa), 1.5 M KCl (–7.04 MPa) or 1.2 M sucrose (–4.85 MPa) were used as hyperosmotic medium and PDA (–0.35 MPa) was used as control medium for non-stressed treatment.

**Figure 2.**
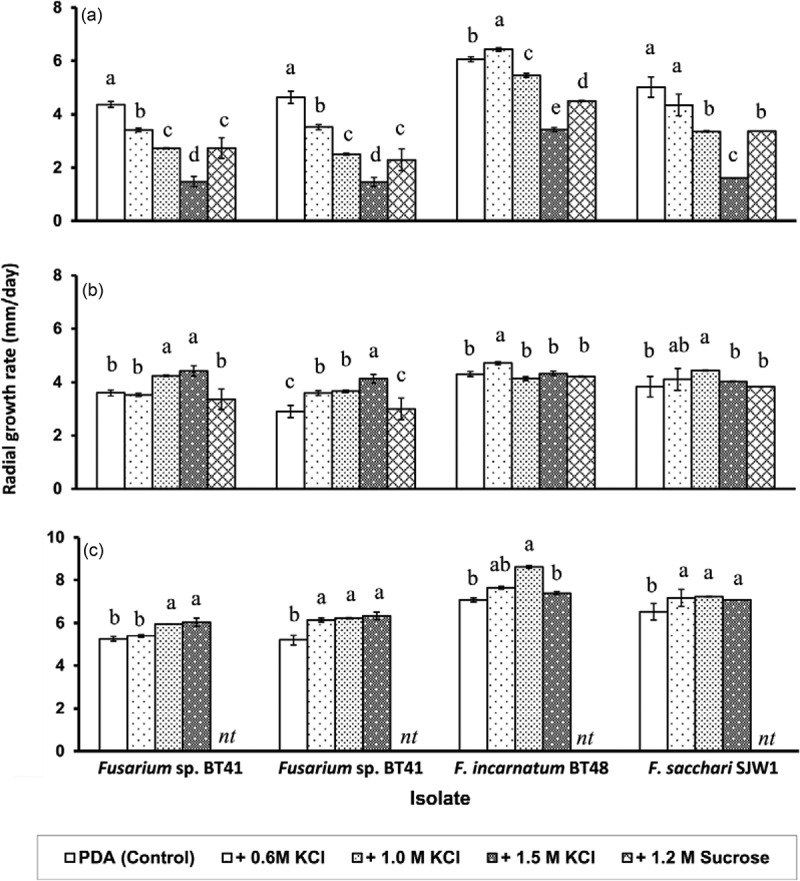
Mycelial growth (mean ± SE) of *Fusarium* isolates from spear rot of oil palm under hyperosmotic stress (a) and during recovery on PMEA (b) and on MM (c). PDA supplemented with 0.6 M KCl (–3.35 MPa), 1.0 M KCl (–4.85 MPa), 1.5 M KCl (–7.04 MPa), or 1.2 M sucrose (–4.85 MPa) were used as hyperosmotic medium and PDA (–0.35 MPa) was used as control medium for non-stressed treatment. Growth during recovery was assessed on a pectin-amended malt extract agar (PMEA) and minimal medium (MM). Graph bars of individual isolate with the same letters are not significantly different (*P* < 0.05) according to Waller–Duncan *K*-ratio *t*-test.

Mycelial growth was suppressed under hyperosmotic stress, although the growth of isolate BT48 was extensive on PDA supplemented with 0.6 M KCl. The growth rate of an isolate on iso-osmotic medium (–4.85 MPa) supplemented with 1.0 M KCl and 1.2 M sucrose was similar (*P* > 0.05). Growth was suppressed in response to increasing KCl concentrations (Figure 1). However, once removed from the hyperosmotic medium and transferred to PMEA or MM, growth recovered rapidly. Mycelia of *Fusarium* sp. grew extensively during recovery from hyperosmotic medium supplemented with 1.0 and 1.5 M KCl. The growth of cultures transferred from sucrose hyperosmotic medium was similar (*P* > 0.05) to that of non-stressed cultures (Figures 1 and 2).

### Virulence of cultures pre-grown on hyperosmotic medium

**Figure 3.**
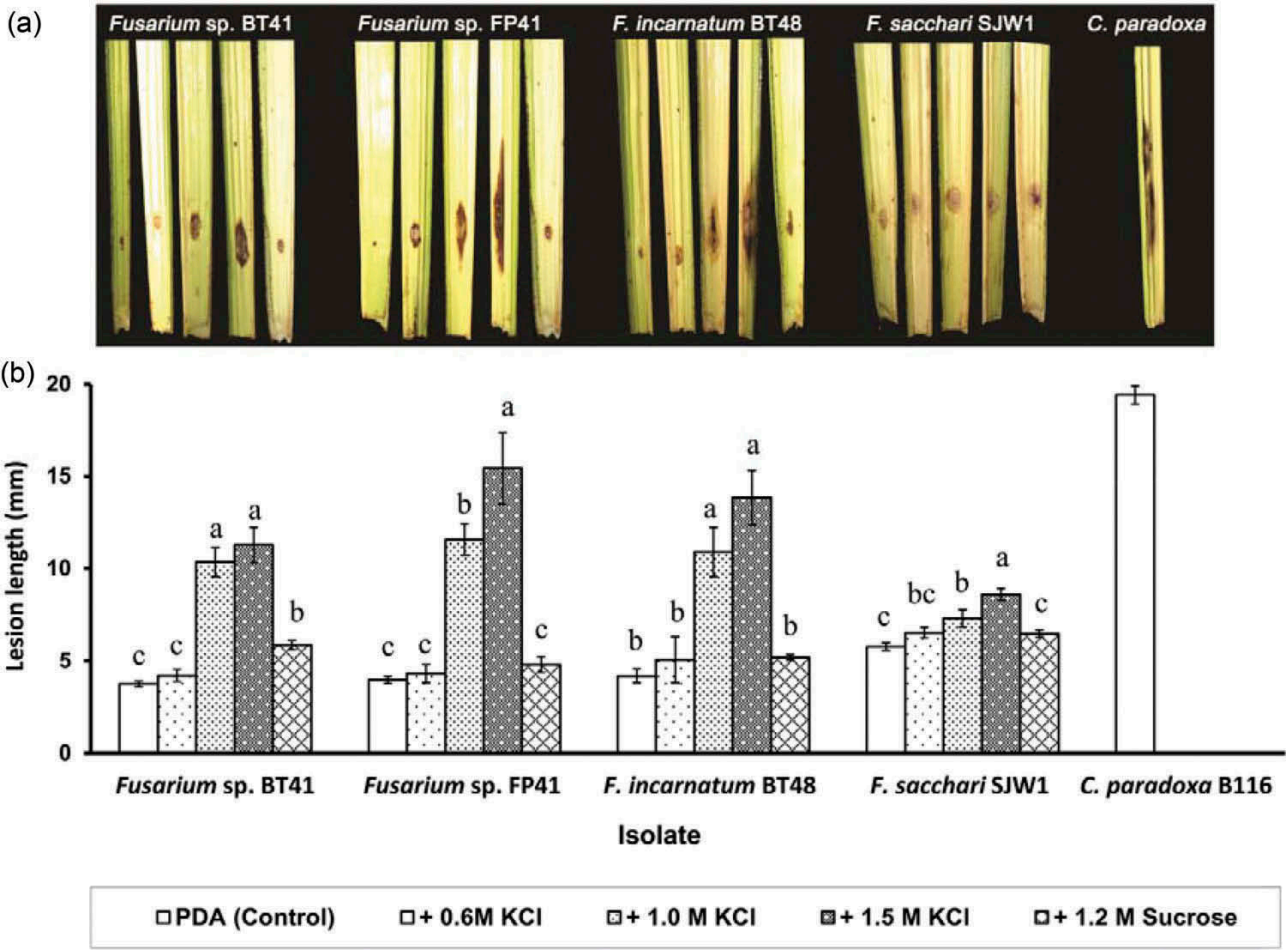
Symptoms (a) and lesion length (mean ± SE) (b) on leaflets of cut spear leaf of oil palm after 4 days inoculated with *Fusarium* isolates precultured for 8 days on PDA supplemented with 0.6 M KCl (−3.35 MPa), 1.0 M KCl (−4.85 MPa), 1.5 M KCl (−7.04 MPa), 1.2 M sucrose (−4.85 MPa), or PDA (−0.35 MPa, control medium for non-stressed treatment). *Ceratocystis paradoxa* B116, a highly virulent isolate, was used as the positive control in virulence assays. The necrotic lesion on each leaflet represents to that represented by the corresponding bar in the graph. Graph bars of individual isolate with the same letters are not significantly different (*P* < 0.05) according to Waller–Duncan *K*-ratio *t*-test.

Lesions on *Fusarium* sp. and *F. incarnatum* precultured on media supplemented with 1.0 and 1.5 M KCl (–4.85 and –7.04 MPa, respectively) were two to four times larger than the small lesions (<5 mm long) on non-stressed cultures. The lesions were typical of CSR symptoms and were less expansive than those caused by strongly virulent *C. paradoxa*. Lesion size was not greatly affected by increased KCl concentrations added to hyperosmotic cultures of moderately virulent *F. sacchari*. Inoculation of sucrose-hyperosmotic cultures resulted in similar (*P* > 0.05) lesion size relative to non-stressed cultures (Figure 3).

### Enzyme activity of Fusarium *isolate during recovery from hyperosmotic stress*

**Figure 4.**
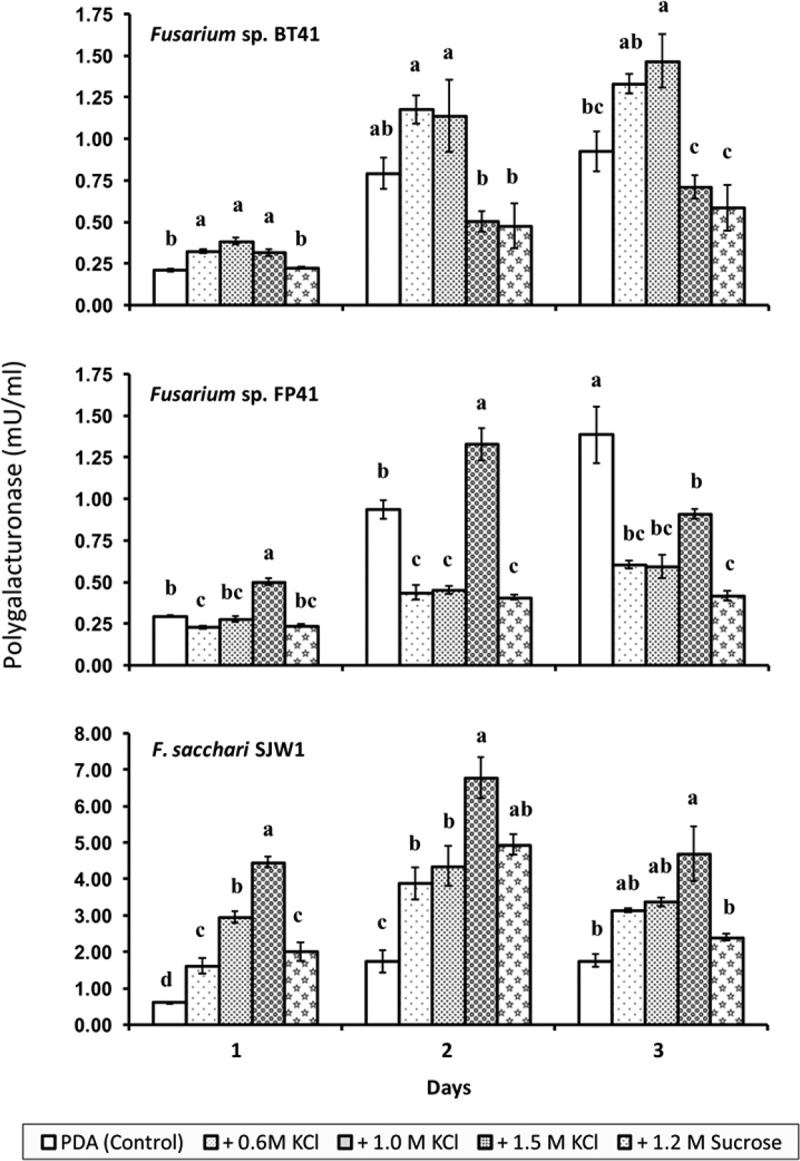
Polygalacturonase activity (mean ± SE) of *Fusarium* spp. precultured for 8 days on PDA supplemented with 0.6 M KCl (–3.35 MPa), 1.0 M KCl (–4.85 MPa), 1.5 M KCl (–7.04 MPa), 1.2 M sucrose (–4.85 MPa), or PDA (–0.35 MPa, control medium for non-stressed treatment) and subsequently were transferred to malt extract broth supplemented with pectin. The *y*-axes are not to the same scale. Graph bars of each incubation period with the same letters are not significantly different (*P* < 0.05) according to Waller–Duncan *K*-ratio *t*-test.

Among two pectinases studied *in vitro*, only PG activity was detected in liquid culture supernatants of *Fusarium* isolates. Increased PG activity was observed in the liquid culture of *Fusarium* sp. isolate BT41 transferred from 1.0 M KCl-hyperosmotic medium and isolate FP41 transferred from 1.5 M KCl-hyperosmotic medium. The isolate of *F. sacchari* secreted a high level of PG activity in liquid culture, and PG activity increased significantly with increasing KCl concentrations during preculturing (Figure 4). *F. incarnatum* isolate did not secrete PG activity during 3 days of incubation in liquid culture (detection limit: 0.05 mU/ml), but PG activity was detected at a low level (<1 mU/ml) after 6 days of incubation.

### Pectinase activity in inoculated spear leaf leaflets

PG activity was detected in leaf tissues inoculated with *Fusarium* sp. and *F. sacchari*, but not those inoculated with *F. incarnatum*. Leaves inoculated with hyperosmotic cultures exhibited 5–32 times higher PG activity than those inoculated with non-stressed cultures (Table 1). Plate assays did not detect PNL activity in inoculated leaves, nor PG or PNL activity in non-inoculated wounded leaves.

**Table 1.** Polygalacturonase activity (mean ± SE) in spear leaf leaflet of oil palm after 4 days inoculated with mycelia of *Fusarium* isolates precultured on hyperosmotic medium (PDA + 1.5 M KCl) or control medium (PDA).

	Polygalacturonase activity (mU/g fresh weight)		
Isolate	PDA + 1.5 M KCl	PDA	*P*^a^
Fusarium sp. BT41	2.19 ± 0.09	0.40 ± 0.02	0.0018
Fusarium sp. FP41	0.94 ± 0.57	0.34 ± 0.01	0.0028
F. incarnatum BT48	nd	nd	–
F. sacchari SJW1	3.73 ± 0.29	0.43 ± 0.04	0.0091
Non-inoculated leaf	nd^b^	nd	–

^a^*P*-values were determined by paired *t*-test.

^b^Not detected (detection limit 0.1 mU/g leaf).

## Discussion

This study demonstrated that *Fusarium* species, which are weak pathogens of oil palm spear rot, can rapidly recover from drought and salt stress. The mycelial growth of *Fusarium* isolates was suppressed under hyperosmotic stress, but growth rapidly recovered when mycelia were transferred to control medium. Suppression of mycelial growth on iso-osmotic medium with KCl (salt/osmotic stress) and sucrose (osmotic stress) was similar, but extensive regrowth was frequently observed during recovery from KCl stress. These results suggest that *Fusarium* species exhibit growth plasticity under hyperosmotic stress and recovery. Prestress treatments with salt showed a greater effect than those with sucrose on growth plasticity.

*Fusarium* sp. and *F. incarnatum* precultured on KCl hyperosmotic medium induced typical CSR lesions on oil palm leaves during the normal growth period, whereas no symptoms developed when non-stressed cultures were inoculated, suggesting an enhanced virulence of the weak pathogenic isolates. The moderately virulent *F. sacchari* showed less enhanced virulence. Cultures transferred from non-ionic stress (sucrose hyperosmotic medium) did not demonstrate a significant increase in virulence.

PG activity was detected in both liquid cultures and inoculated leaves. No activity of PNL was detected in liquid cultures of the *Fusarium* isolates. All isolates except *F. incarnatum* BT48 secreted PG activity in both liquid cultures and inoculated leaves. Because no PG activity was detected in non-inoculated leaves, PG detected in inoculated leaves was considered to be exclusively of fungal origin. Leaves inoculated with hyperosmotic cultures of *Fusarium* sp. and *F. sacchari* showed significantly increased PG activity, indicating the potential role of pectinase in the increased rotting activity of these CSR pathogens. Our results also support previous findings that species in the *G. fujikuroi* complex secrete PGs during infection and play a crucial role in plant tissue penetration and colonisation (Mariotti et al. 2009).

The inoculation of oil palm leaves with *F. incarnatum* precultured on 1.5 M KCl-hyperosmotic medium resulted in lesions that were four times larger than those of non-stressed cultures, but no PG activity was detected in lesion homogenates, nor in liquid culture secretions during 3 days of recovery growth. PG activity did occur at a low level after 6 days of incubation. Most likely, *F. incarnatum* does not secrete PG activity during early growth; thus, PG can be considered to play a less important role in increased rotting activity by this fungus.

These findings suggest that *Fusarium* sp., *F. incarnatum*, and *F. sacchari* exhibit an adaptive physiological plasticity to hyperosmotic stress (osmoadaptation) that results in enhanced virulence. Osmoadaptation involves both physiological and genetic manifestations of adaptation to a low-water environment (Galinski 1995). Osmoadaptation in fungi mainly through the action of the high osmolarity glycerol mitogen-activated protein kinase (MAPK) signalling pathway leads to a response necessary for adapting and surviving hyperosmotic environments (Duran et al., 2010). The osmoregulation MAPK pathway is well conserved in fungi. The inactivation of Hog1 orthologs increases sensitivity to osmotic stress and attenuates the virulence of *Beauveria bassiana* (Zhang et al. 2009), *Cryphonectria parasitica* (Park et al. 2004), *Fusarium graminearum* (Zheng et al. 2012) and *Metarhizium acridum* (Jin et al. 2012), and *Ustilaginoidea virens* (Zheng et al. 2016). The osmoadaptative response of *F. verticillioides* is also coupled to the marked induction of *FUM1* gene expression, a gene required for fumonisin biosynthesis (Marín et al. 2010). To the best of our knowledge, this is the first study to provide evidence that osmotic stress is associated with enhanced virulence of the weak plant-pathogenic *Fusarium* spp. An association between osmotic stress and a slightly increased virulence has also been observed in the entomopathogenic fungus *Metarhizium anisopliae* (Rangel et al. 2008).

The enhanced virulence of CSR fungi confirmed in this study may occur in the field. The leaf water potential of oil palm seedlings subjected to drought for 24 days was –3.6 MPa (Suresh et al. 2010). In this study, cultures recovering from –3.35 MPa showed a slight increase in virulence. During the dry season, oil palms grown in peat lowland areas suffer from salt and osmotic stress (Mutert et al. 1999). CSR is prevalent in oil palms in peat lowland areas of Sumatra and Kalimantan (unpublished observation). With global climate changes, oil palm in the tropics and subtropics will more likely experience more intense and longer drought stress (Paterson et al. 2013). Further study is needed to confirm the *in planta* responses of oil palm leaf-colonising *Fusarium* spp. following recovery from salt stress. In *F. verticillioides*, a pathogen of maize stalk rot, environmental stresses including drought have been considered to induces changes in host physiology and to trigger the switch from an asymptomatic endophytic to a virulent pathogenic lifestyle (Ridenour et al., 2014). Enhanced virulence of *Fusarium* spp. following post-stress recovery, as revealed in this study, together with the evidence about predisposing role of environmental stresses on oil palm susceptibility (Alvarado et al. 1996; Sterling & Alvarado 1996; Suwandi & Kondo 2012) highlight the risk that CSR will arise in the future as a consequence of global climate changes.
